# The O-antigen negative ∆*wbaV* mutant of *Salmonella enterica* serovar Enteritidis shows adaptive resistance to antimicrobial peptides and elicits colitis in streptomycin pretreated mouse model

**DOI:** 10.1186/s13099-015-0070-4

**Published:** 2015-09-03

**Authors:** Sangeeta Jaiswal, Niladri Bhusan Pati, Manupriyam Dubey, Chandrashekhar Padhi, Prakash Kumar Sahoo, Shilpa Ray, Aryashree Arunima, Nirmal Kumar Mohakud, Mrutyunjay Suar

**Affiliations:** KIIT School of Biotechnology, KIIT University, Bhubaneswar, Odisha 751024 India; Kalinga Institute of Medical Sciences, KIIT University, Bhubaneswar, Odisha 751024 India

**Keywords:** *S*. Enteritidis, Lipopolysaccharide, OAg-negative, *wbaV* gene, Antimicrobial peptides, Virulence, OAg biosynthesis

## Abstract

**Background:**

*Salmonella enterica* serovar Enteritidis, the most common cause of human gastroenteritis, employs several virulence factors including lipopolysaccharide (LPS) for infection and establishment of disease inside the host. The LPS of *S. enterica* serovar Enteritidis consists of lipid A, core oligosaccharide and O-antigen (OAg). The OAg consists of repeating units containing different sugars. The sugars of OAg are synthesized and assembled by a set of enzymes encoded by genes organized into clusters. Present study focuses on the effect of deletion of genes involved in biosynthesis of OAg repeating units on resistance to antimicrobial peptides and virulence in mice.

**Methods:**

In the present study, the OAg biosynthesis was impaired by deleting *tyv, prt* and *wbaV* genes involved in tyvelose biosynthesis and its transfer to OAg. The virulence phenotype of resulting mutants was evaluated by assessing resistance to antimicrobial peptides, serum complement, adhesion, invasion and in vivo colonization.

**Results:**

Deletion of the above three genes resulted in the production of OAg-negative LPS. All the OAg-negative mutants showed phenotype reported for rough strains. Interestingly, Δ*wbaV* mutant showed increased resistance against antimicrobial peptides and normal human serum. In addition, the Δ*wbaV* mutant also showed increased adhesion and invasion as compared to the other two O-Ag negative mutants Δ*tyv and* Δ*prt*. In vivo experiments also confirmed the increased virulent phenotype of Δ*wbaV* mutant as compared to Δ*prt* mutant.

**Conclusion:**

OAg-negative mutants are known to be avirulent; however, this study demonstrates that certain OAg negative mutants e.g. ∆*wbaV* may also show resistance to antimicrobial peptides and cause colitis in *Streptomyces* pretreated mouse model.

## Background

**Fig. 1:**
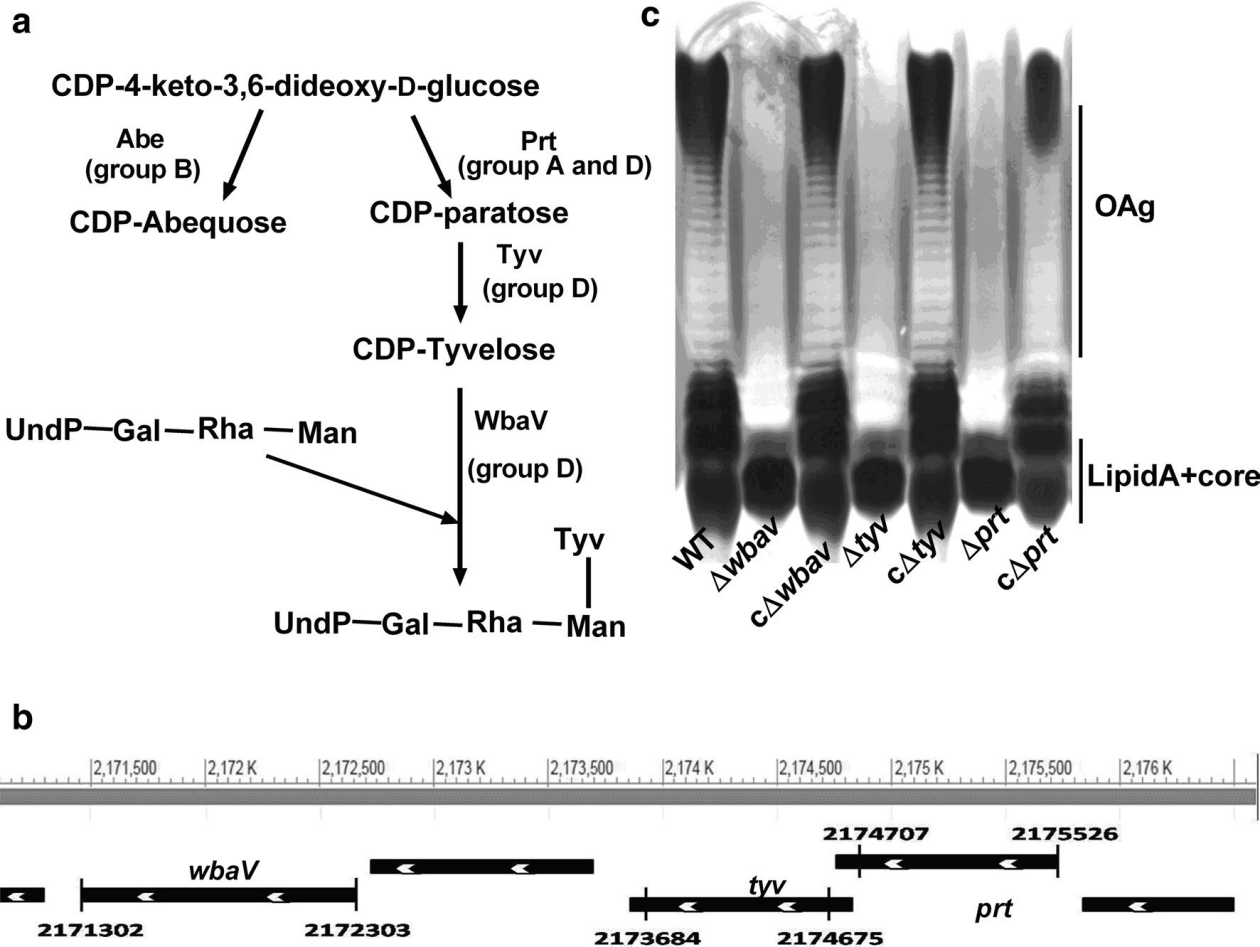
Schematic presentation of final steps of dideoxy sugar biosynthesis, location of genes on chromosome and LPS profile of wild-type *S.* Enteritidis and its isogenic mutants. **a** CDP-paratose is synthesized by CDP-4-keto-3,6-dideoxy-d-glucose by the action of prt which is epimerized by Tyv to tyvelose. After synthesis, tyvelose is transferred to OAg backbone of mannose (Man), rhamnose (Rha) and galactose (Gal) by WbaV. CDP-abequose is also synthesized from same CDP sugar intermediate as CDP-paratose. **b** Location of genes on chromosome. The regions enclosed by the *black bar* were deleted with the help of lambda *red* recombinase system. *White arrow* indicate that genes are located on negative strand. **c** LPS from wild-type *Salmonella* as well as the mutants were isolated using the protocol described in materials and methods and separated on polyacrylamide gel electrophoresis (PAGE) gel using tricine-SDS buffer system. LPS were visualized by silver staining. **c**∆ indicates the mutants are complemented with the corresponding genes

*Salmonella* are Gram-negative, intracellular bacteria that cause diseases ranging from acute gastroenteritis to typhoid fever, posing a significant threat to public health globally. Infections with non typhoidal serovars of *Salmonella enterica,* predominantly *S. enterica* serovar Enteritidis (*S.* Enteritidis) and *S. enterica* serovar Typhimurium (*S*. Typhimurium) are more frequent and occur in both developing and industrialized nations [1]. *Salmonella* employs a number of virulence factors to successfully colonize and replicate inside the host. The most critical virulence determinants of *Salmonella* infections are *Salmonella* pathogenicity islands (SPI), of which SPI-1 and SPI-2 play crucial role in invasion and intracellular replication respectively. Apart from these, numerous additional virulence factors such as pilli or fimbria [2], flagella [3], lipopolysaccharide (LPS) [4] etc. are required for a successful infection. Among these factors, role of LPS in *Salmonella* virulence has been established by several studies [5–7]. LPS is a major structural component of the outer membrane of all the Gram-negative bacteria and plays a critical role in the bacterial pathogenesis. It consists of three distinct domains: lipid A, core oligosaccharide (OS) and O-antigen polysaccharide (OAg). The different components of LPS interact with different parts of host cells and contribute to bacterial pathogenesis. For example, different sugars of outer core interact with epithelial cells, whereas, lipid A interacts with the TLR4, a surface receptor of immune cells. While lipid A and core OS structures are fairly conserved, the OAg is highly variable, leading to serological specificity among Gram-negative bacteria. OAg is a modular assembly of oligosaccharide units that varies with respect to the sugar composition and number of their modal repeats [8]. Typically, in all the Gram-negative bacteria, this region comprises of 16 to more than 100 repeats of oligosaccharide units containing 4–6 monosaccharides each. Bacteria lacking OAg are called rough. The repeating unit of OAg of *Salmonella* mainly consists of three hexose sugars namely mannose, rhamnose, galactose and one dideoxy hexose as the fourth component [9]. Mannose, rhamnose and galactose form the backbone of the OAg and are conserved across different serovars of *Salmonella*. The dideoxy sugar linked (α-1, 3) to mannose residue varies among different serovars. Based on the agglutination by antibodies against different O-antigens, *Salmonella* have been grouped into six serogroups namely A, B, C1, C2, D and E. The group A (e.g. *S*. Typhi) contain paratose (3,6-dideoxy-d-*ribo*-hexose), group B (*S.* Typhimurium) has abequose, whereas group D (e.g. *S*. Enteritidis) contain tyvelose (3,6-dideoxy-d-*arabino*-hexose) as fourth component of OAg repeating unit [10]. A schematic representation of the biosynthetic pathway of these sugars is shown in Fig. 1a. In *S.* Enteritidis, enzyme paratose synthase (Prt, formerly known as RfbS), synthesizes paratose, which is further epimerized by the enzyme CDP-tyvelose-2-epimerase (Tyv, formerly known as RfbE), to tyvelose which is then transferred to OAg repeating unit by tyvelosyl transferase (WbaV, formerly known as rfbV) (Fig. 1a). The genes responsible for OAg biosynthesis are generally found on the chromosome as an OAg gene cluster and genetic variation in this cluster reflects the structural variations of OAg across Gram-negative bacteria. This gene cluster encodes proteins that can be further categorized into three groups [11]. First group involve proteins that synthesize nucleotide sugar precursors. Proteins of the second group are mainly glycosyl transferases (GTase) that build sequentially, the OAg-repeating unit on the carrier lipid, undecaprenyl phosphate (UndP). The third group of enzyme is mainly OAg processing enzyme involved in the polymerization and translocation of OAg across the membrane. The enzymes, Tyv and Prt are involved in biosynthesis of CDP-sugars whereas WbaV is a GTase. The location of the above genes on chromosome has been depicted in Fig. 1b.

Deletion of the genes involved in nucleotide diphosphate (NDP) sugar biosynthesis renders bacteria unable to synthesize complete OAg [12]. For example, deletion of *galE* or *pmi* genes involved in NDP sugar biosynthesis, results in the truncation of LPS which can be further restored via trans-complementation [12–14]. Since, both *prt* and *tyv* are involved in CDP-sugar biosynthesis [15, 16], their deletion could possibly affect OAg biosynthesis. OAg biosynthesis and assembly is a highly co-ordinated process and is accomplished step by step. Interference at any step may not only affect the OAg biosynthesis but also other cellular processes as sugar intermediates are synthesized from precursors obtained from common metabolic pathways [16]. In the present study, we have blocked tyvelose biosynthesis and its transfer to OAg repeating units by gene deletion (*tyv*, *prt* and *wbaV*) and investigated their effect on *S.* Enteritidis virulence. Interestingly, we found that deletion of tyvelosyl transferase gene (*wbaV*) resulted in increased resistance against antimicrobial peptides (AMPs) and decreased degree of attenuation in the rough strain of *S.* Enteritidis as compared to other OAg-negative strains.

## Results

### Deletion of genes involved in OAg biosynthesis from the genome of *S*. Enteritidis results in the production of OAg-negative LPS

In order to investigate the effect of deletion of genes involved in biosynthesis of OAg, the Δ*wbaV,* Δ*tyv*, and Δ*prt* gene were deleted from the genome of *S.* Enteritidis and LPS from the respective deletion mutant was analyzed by SDS PAGE and silver staining. It was observed that the strains were unable to synthesize OAg repeating units (Fig. 1b). However, the synthesis of lipid A and core region remained unaffected. To prove that the deletion of genes had no polar effect on downstream genes, mutant strains were complemented with their respective genes with the help of plasmid listed in Table 1. Upon complementation, all the strains synthesized complete OAg repeating unit (Fig. 1c).

**Table 1:** Strains and plasmids used in this study

Strain/plasmid	Description	Resistance	References
*S.* Enteritidis	Wild-type	Sm^r^	[42]
Δ*wbaV*	*wbaV*::*aphT*	Sm^r^, Km^r^	Present study
Δ*tyv*	*tyv* ::*aphT*	Sm^r^, Km^r^	Present study
Δ*prt*	*prt::aphT*	Sm^r^, Km^r^	Present study
Δ*invC*	SPI1^−^ (*invC*::*aphT)*	Sm^r^, Km^r^	Present study
pKD46	bla PBAD gam bet exo pSC101 oriTS	Amp^r^	[38]
pKD4	bla FRT aph FRT PS1 PS2 oriR6K	Km^r^	[38]
pCH112	*hilA* ORF cloned into P*BAD*/*myc*-His; *ori*pBR322	Amp^r^	[39]

### OAg-negative mutants showed significant impairment in the motility

**Fig. 2:**
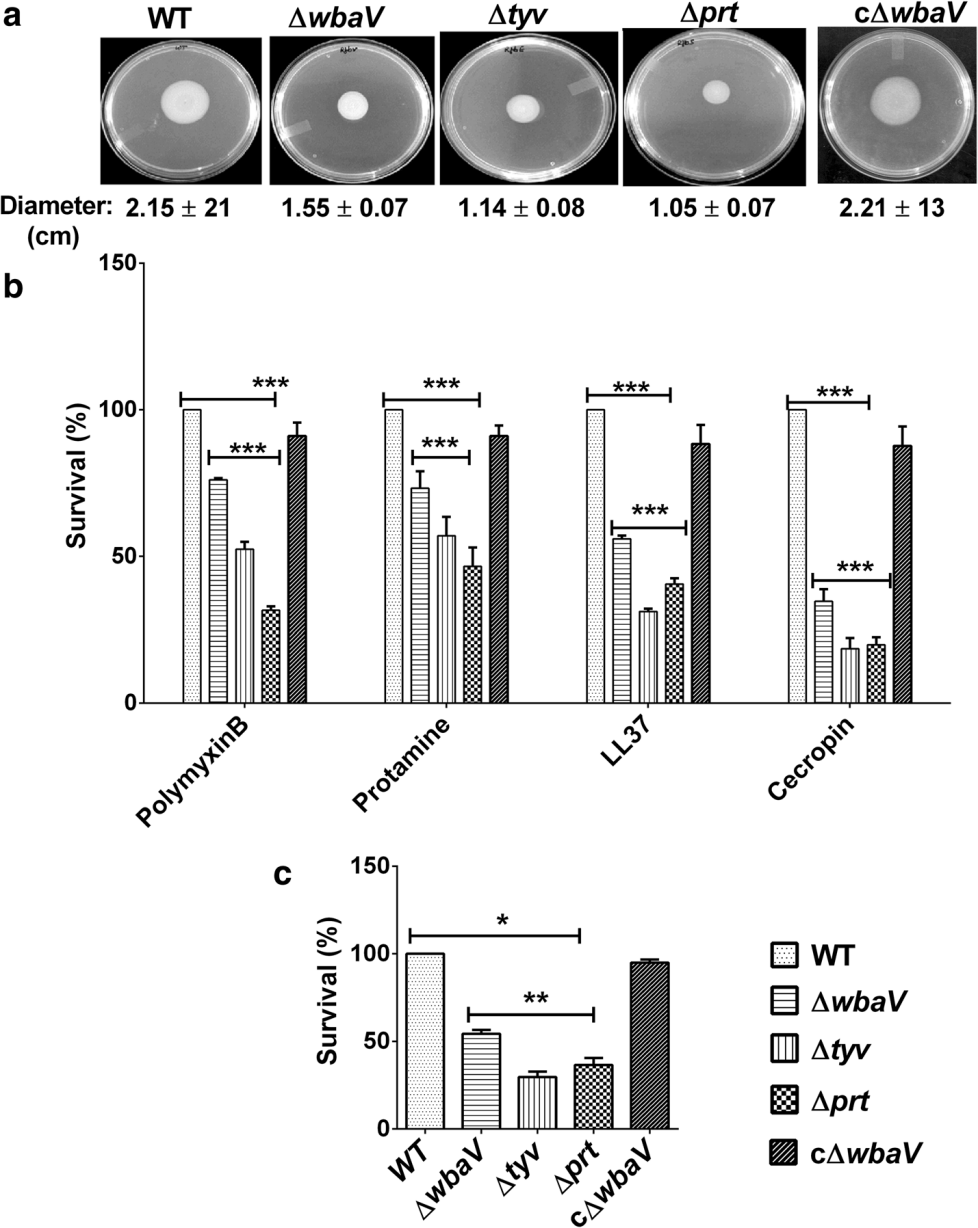
Assessment of motility and sensitivity to antimicrobial peptides and serum. **a** Wild-type *Salmonella* and its isogenic mutants were placed at the center of the agar plate containing 0.3 % agar and incubated for 8 h. Diameter of growth zone was measured in centimeter (cm). Experiments were performed in triplicates at three independent occasions. **b** For AMP sensitivity assay, approximately 1 × 10^7^ CFU/ml bacteria in PBS were incubated with or without antimicrobial peptides (1 µg/ml polymyxin B, 5 µg/ml protamine, 10 µg/ml LL37, and 2 µg/ml cecropin) for 1 h at 37 °C. The number of surviving bacteria was determined by plating serial dilutions on LB agar plates and survival percentage was calculated. Statistical analysis was performed using two-way ANOVA. **c** For analysis of serum resistance 1 × 10^7^ CFU/ml of bacteria was incubated with or without 50 % of normal human serum and serial dilutions were plated to enumerate the number of surviving bacteria. Statistical analysis was performed using student *t*-test. The survival of Δ*prt* and Δ*tyv* mutants was compared with that of Δ*wbaV*. Level of significance is indicated by *asterisks* (*P < 0.05, **P < 0.01, ***P < 0.001), *ns* not significant. *Error bar* indicate the standard deviation of three independent experiments

Bacterial strains lacking OAg are known to be less motile as compared to those with long OAg repeating units. To confirm if the OAg-negative mutants used in this study were also less motile, the motilities of the mutants were analyzed by soft agar assay. All the OAg-negative mutants were found to be less motile as compared to the wild-type and showed almost two fold reduction in their motility (Fig. 2a).

### OAg-negative mutants showed differential sensitivity to AMPs and complement mediated killing

The structure and composition of LPS is known to affect the susceptibility of bacterial strains to various cationic AMPs [17, 18]. Therefore, we evaluated the sensitivity of these mutants to different cationic AMPs. The survival efficiency of the mutants was compared to that of the wild-type (normalized to 100 percent). All the mutants showed increased susceptibility to AMPs tested in this study as compared to the wild-type. Interestingly, the Δ*wbaV* mutant showed significantly increased resistance to all the AMPs as compared to other OAg-negative mutants (*P* < 0.001), which was reduced up to the level of wild-type upon complementation (Fig. 2b).

To further characterize the mutants, the sensitivities of mutants to complement mediated killing was determined. All the OAg-negative mutants were found to be more susceptible to killing by serum as compared to wild-type strain. The OAg-negative strains showed 50 % or less survival in serum when compared to the wild-type (normalized to 100 %). The Δ*wbaV* mutant, however, showed significantly increased survival (*P* < 0.05–0.01) as compared to other two OAg-negative mutants (Δ*prt* and Δ*tyv*) (Fig. 2c).

### The Δ*wbaV* mutant showed increased adhesion and invasion efficiencies

**Fig. 3:**
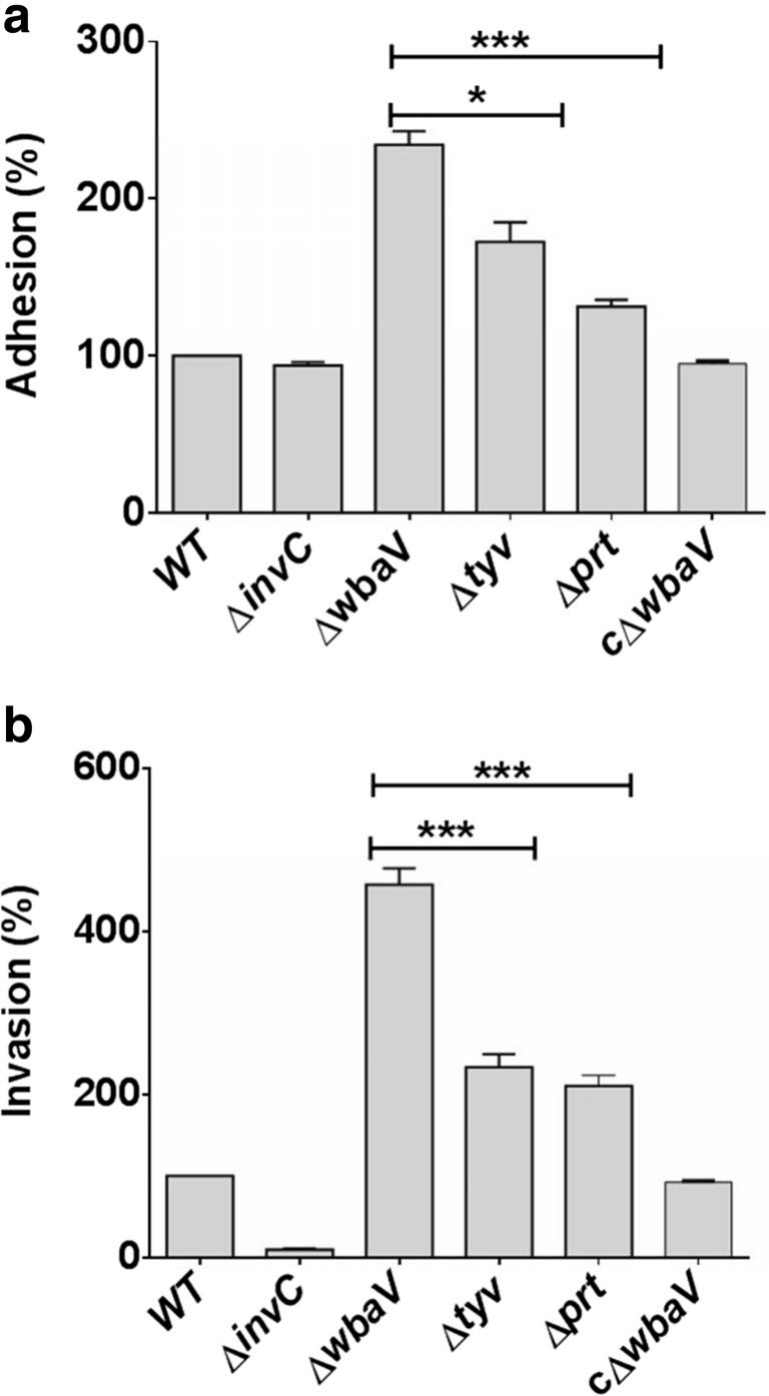
Attachment and invasion of *S.* Enteritidis wild-type and its isogenic mutants to HCT116 cell line. **a** HCT116 cells were seeded at the density of 2 × 10^5^ cells per well and bacterial strains were infected at the MOI of 10. Infection was performed on ice followed by incubation at 4 °C for 30 min. Non adherent bacteria were removed by washing and number of adhering bacteria was enumerated by plating serial dilution. **b** For invasion, HCT116 cells were seeded at the density of 2 × 10^5^ cells per well and bacterial strains were infected at the MOI of 10. Number of invading bacteria was determined through standard gentamicin protection assay. SPI1 negative strain Δ*invC* served as an experimental control. Experiments were performed in triplicates. Percentage of adhesion, invasion and uptake of mutant was compared to wild-type (normalized to 100). *Error bar* indicate the standard deviation of three independent experiments. Statistical analysis was performed using *t* test. The percentage of adhesion and invasion replication of Δ*prt* and Δ*tyv* was compared with that of Δ*wbaV* and level of significance is indicated by *asterisks* (*P < 0.05, **P < 0.01, ***P < 0.001), *ns* not significant

To study the effect of deletion of above genes in adhesion and invasion, the ability of different OAg mutants to adhere and invade HCT-116 cells was compared. OAg- negative mutants attached and invaded HCT-116 cells more efficiently as compared to the parental strain. Among all the three mutants, Δ*wbaV* mutant showed the highest rate of adhesion (~2.5-fold) and invasion (~4.5-fold) when compared with the wild-type (Fig. 3a, b) and the complemented strain showed adhesion and invasion similar to wild type. However, variations in the efficiency of adhesion and invasion among mutants were observed. The Δ*wbaV* mutant showed significantly increased adhesion and invasion when compared with other two OAg- negative mutants (*P* < 0.05–0.001).

### *S.* Enteritidis Δ*wbaV* mutant showed increased systemic load as compared to Δ*prt* mutant in C57BL/6 mice

**Fig. 4:**
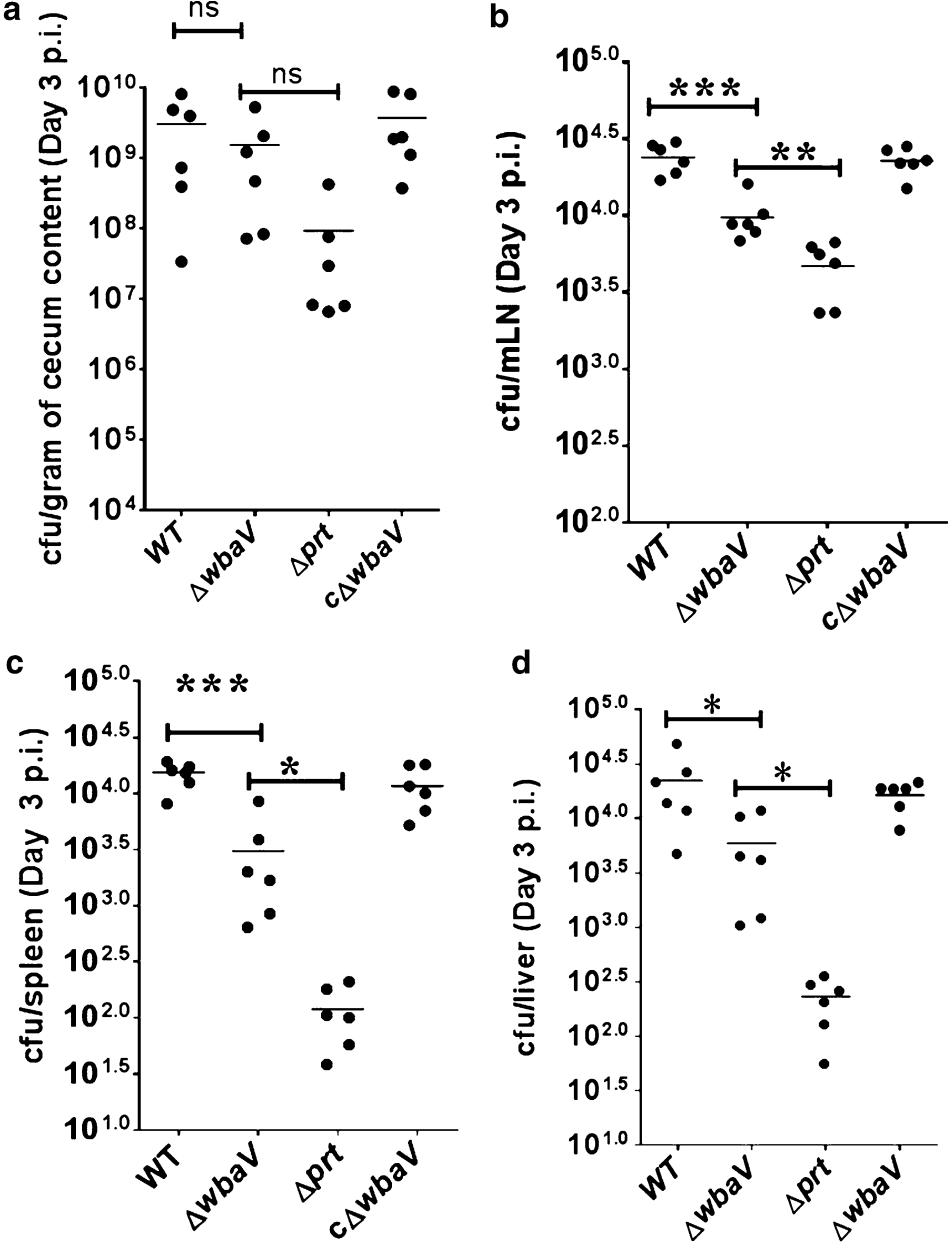
Colonization of cecum, mLN, spleen and liver of C57BL/6 mouse by *S.* Enteritidis and OAg-negative mutants: Streptomycin pretreated mice were infected with respective bacterial strains through oral route of administration. Mice were sacrificed on 3 days p. i. and cecum (**a**), mLN (**b**), spleen (**c**) and liver (**d**) were collected and CFU in each organ were determined. Statistical significance is indicated by *asterisks* (*P < 0.05, **P < 0.01, ***P < 0.001), *ns* not significant

**Fig. 5:**
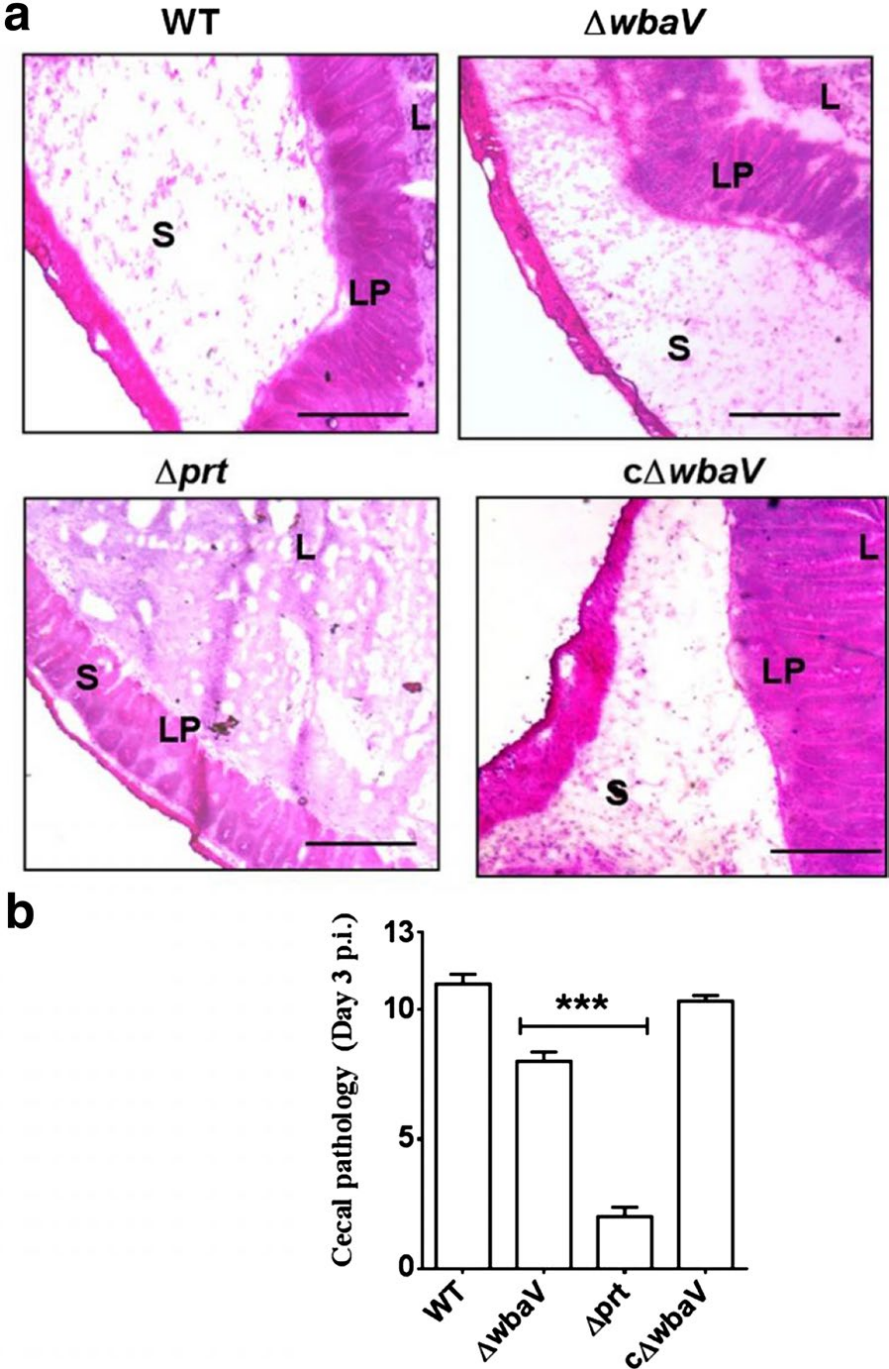
Histopathological evaluation of cecum from mouse infected with *S.* Enteritidis and OAg-negative mutants. HE stained sections of cryoembeded cecal tissue showing cecal inflammation (**a**) and respective pathoscore (**b**) was determined by semi quantitative method as described in “Methods”. *S* submucosal edema, *LP* lamina propria, *L* lumen. *Bars* 200 μm

To understand the effect of deletion of above genes on virulence of Δ*wbaV* mutant in vivo, C57BL/6 mice were infected with wild-type, Δ*wbaV* and Δ*prt* mutants. In a 3 days p. i. experiment, the cecal colonization of Δ*wbaV* mutant was comparable to wild-type, whereas the Δ*prt* mutant colonized less efficiently than Δ*wbaV* mutant and the wild-type (Fig. 4a), however, the differences were not statistically significant. On the contrary, the pathogen load in the mesenteric lymph node (mLN) varied between both the mutants. The CFU of Δ*wbaV* mutant recovered from mLN was significantly (*P* = 0.0085) higher than Δ*prt* mutant (Fig. 4b). Similarly, the bacterial load in spleen and liver was also found to be significantly higher in case of Δ*wbaV* mutant as compared to Δ*prt* mutant (*P* < 0.05) (Fig. 4c, d). Notably, the Δ*wbaV* mutant showed reduced colonization in mLN, spleen and liver as compared to the wild-type. Further, histopathological evaluation was carried out to compare the cecal inflammation between the mutants and wild-type. The Δ*wbaV* mutant was found to induce moderate cecal inflammation whereas Δ*prt* mutant was unable to induce inflammation in the C57BL/6 mice (Fig. 5a). The cecal pathology was assessed by a semi-quantitative scoring method as described in materials and methods. The day 3 p. i. experiment showed a significant difference in the cecal pathology of the mice infected with Δ*prt* mutant (pathoscore: 8) and Δ*wbaV* mutant (pathoscore: 2) as compared to the wild-type (P < 0.01) (Fig. 5b). The phenotype of complemented Δ*wbaV* strain was similar to wild type.

## Discussion

LPS forms the major component of the outer membrane of Gram-negative bacteria, though ubiquitously present, its structure and composition varies among species. The most variable part of LPS is the OAg chain, consisting of repeating unit of four to six sugars which forms the basis of serological typing. The serovars of *Salmonella* mainly differ in the presence of dideoxy sugars in OAg repeating unit. In the present study, we have investigated the effect of deletion of three OAg biosynthetic genes on virulence of *S.* Enteritidis.

Deletion of each gene targeted in this study resulted in the production of OAg-negative LPS. Deletion of *prt* gene blocks the synthesis of CDP-paratose which cannot be further epimerized to tyvelose by *tyv* gene product; as a result Δ*prt* mutant cannot synthesize OAg. However, in Δ*tyv* mutant, epimerization of CDP-paratose into tyvelose was hampered due to deletion of *tyv* gene but the synthesis of CDP-paratose remains unaffected. It has been reported earlier, that tyvelosyl transferase of *S.* durban has an affinity for CDP-paratose and can transfer it to OAg [10]. Similarly, the group A, B and D glycosyl transferases are able to transfer paratose, abequose and tyvelose to OA [19, 20]. However, Osborn and Weiner, 1968 reported that glycosyl transferase of B1 has more specificity for its natural substrate [21]. In this study, the ability to synthesize complete OAg was restored after complementation indicating that rough phenotype of Δ*tyv* mutant was not due to polar effect. Therefore, the inability of Δ*tyv* mutant to synthesize complete OAg suggests that the tyvelosyl transferase (*wbaV*) of *S*. Enteritidis is unable to transfer paratose to OAg repeating unit. Further, deletion of *wbaV* gene also resulted in the synthesis of OAg-negative LP*S*, suggesting that, the addition of dideoxy sugar to the OAg sugar backbone is crucial for the complete biosynthesis of OAg. Similar finding was reported by Hong et al. [22].

It is already established in previous studies, that OAg-negative mutants are more susceptible to killing by serum [23, 24], and cationic AMPs such as polymyxin B [23–25]. We also reported the similar findings, and showed that all the mutants were more susceptible to killing by serum and AMPs as compared to wild-type. Earlier reports also demonstrated that the OAg-negative strains have increased efficiency of adhesion and invasion into non phagocytic cells [25, 26]. Our data correlates well with these findings where all the OAg-negative mutants were adhering and invading more efficiently into HCT-116 cells as compared to the wild-type. It was reported that the outer core sugars of LPS are crucial for the interaction of the bacteria with the epithelial cells, [27, 28] therefore, in the absence of OAg, early interaction of core sugars with epithelial cells is facilitated contributing to increased adhesion. Further, increased invasion of O-Ag negative mutants can be explained on the basis of the model proposed by Holzer et al. [25]. The model states that long and very long OAg interferes with the interaction of type III secretion complex with the host cell membrane and impairs the translocation of effector proteins from bacteria to host cell [25]. Thus, in the absence of OAg, this interference is abolished resulting in the increased efficiency of invasion. In this study, mutants showing increased invasion also showed increased adhesion. Therefore, we propose that increased adhesion efficiency along with the enhanced type III secretion system functionality contributed to the significantly increased invasion efficiency to OAg-negative mutants. Notably, in spite of increased invasiveness, the O-Ag negative mutants did not show increased colonization of cecum suggesting, invasiveness alone does not determine the colonization capacity of *Salmonella*.

In the present study, the Δ*wbaV* mutant showed increased resistance to AMPs and serum as compared to other OAg-negative mutants. It also showed increased adhesion and invasion as compared to other OAg-negative mutants. To support the in vitro data, mouse experiments were carried out with wild-type, Δ*wbaV* and Δ*prt* mutants of *S.* Enteritidis. In, in vivo experiments, no significant difference was observed in cecal colonization of both the mutants, however, the bacterial count of Δ*wbaV* mutant in mLN was significantly higher than the Δ*prt* mutant. Similarly, Δ*wbaV* mutant showed increased colonization in spleen and liver as compared to Δ*prt* mutant. Further, Δ*wbaV* mutant elicited colitis while Δ*prt* mutant was unable to cause colitis. These findings suggest that Δ*wbaV* mutant is less attenuated as compared to Δ*prt* mutant, which can be explained on the basis of its increased resistance to AMPs and serum.

All the mutants in the present study showed phenotypes similar to an OAg negative strains which are generally avirulent [26]. However, our in vitro and in vivo data demonstrates that the Δ*wbaV* mutant, in spite of being OAg negative, showed a virulent phenotype. Similar finding was reported by Ilg et al. [26] where mutant of *S.* Typhimurium lacking O-Ag side chains elicited acute intestinal inflammation. The ability to cause colitis is determined by several factors including stimulation of host immune response which depends on the interaction of LPS and other pathogenesis related patterns with host cell receptors. Modification of LPS is one of the mechanism through which bacteria attain resistance to antimicrobial peptides. Further, LPS modification also affects the interaction of bacteria with TLRs affecting the signaling pathways which are responsible for production of inflammatory cytokines. Recently, Spahich et al. [29] highlighted an important relation between LPS biosynthesis and outer membrane composition [29]. They found that deletion of specific LPS biosynthetic genes downregulates the transcription of an outer membrane protein, Hap and block its insertion into outer membrane [29]. Further, their study also demonstrated that glycosyltransferase activity but not LPS structure affected the insertion of Hap into outer membrane. Their finding suggests that deletion of LPS biosynthetic genes may affect gene expression through unknown mechanism. According to Tam and Missiaka [30], the intermediate compounds of LPS biosynthesis may exert feedback control on LPS biosynthetic pathway which is regulated by σ^E^ response. σ^E^ belongs to the family of extracytoplasmic factors and serves as an alternative sigma factor encoded by *rpoE* gene. Earlier studies have reported that changes in outer membrane protein as well as lipopolysaccharide structure induce σ^E^ dependent response [30, 31]. In *Escherichia coli*, σ^E^ factor is also involved in transcription of several genes of LPS biosynthetic pathway (for example, *rfaD*, *lpxD*, *lpxA* and *ecfA*) [32]. *rpoE* gene is also known to control the alteration in the structure of both lipid A and core oligosaccharide [33]. LPS remodeling has been depicted as a survival strategy employed by bacteria for adaptation to various environmental changes [34]. In present study, the gene *wbaV* is a glycosyltransferase and deletion of which is expected to result in the accumulation of CDP-tyvelose (intermediate sugar) in the cytoplasm. Also, the ∆*wbaV* mutant showed increased resistance to antimicrobial peptides and serum which most commonly is observed in case of LPS modifications particularly in lipidA modification [18, 35–37]. Based on the phenotype observed in ∆*wbaV* mutant, it can be hypothesized that deletion of *wbaV* gene not only affects the OAg biosynthesis but also certain other unknown cellular process which contributes to the increased resistance to antimicrobial peptides and decreased attenuation in mice.

## Conclusion

To conclude, we found that the deletion of *wbaV* gene conferred an adaptive resistance to antimicrobial peptides resulting in less attenuation in mice as compared to other OAg-negative mutants. Present study demonstrates that certain OAg negative strains can trigger colitis in mouse. Thus, increased resistance to AMPs might be one of the mechanisms employed by *S.* Enteritidis to compensate the loss of OAg side chains for retaining its pathogenicity.

## Methods

### Bacterial strains and growth conditions

The bacterial strains and plasmids used in this study are listed in Table 1. Wild-type *S.* Enteritidis P125109 strain and its isogenic mutants were grown in Luria–Bertani (LB) medium (10 g/l Bacto tryptone, 5 g/l Bacto yeast extract, 5 g/l NaCl, HiMedia, India), at 37 °C. For invasion experiments, bacterial strains were grown in SPI1 inducing environment (LB containing 0.3 M NaCl) at 120 rpm. Antibiotics in growth media were used at the following concentrations: ampicillin, 100 µg/ml; kanamycin, 50 µg/ml; and streptomycin, 50 µg/ml.

### Generation of deletion mutant and complementation

Gene deletion was performed using one-step inactivation method as described previously [38]. Briefly, the Tn5 neomycin phosphotransferase gene (aphT) (conferring resistance to kanamycin) in template plasmid pKD4 was PCR amplified using primers listed in Table 2. The primers were designed such that the overlapping coding sequences of adjacent genes do not get deleted and thus the expression of adjacent genes was not affected. The deleted regions have been shown in Fig. 1c. *S.* Enteritidis wild-type strain harboring plasmid pKD46 was transformed with PCR amplified product and transformants were screened on LB agar plates supplemented with kanamycin. Gene knock-out was confirmed by PCR using kanamycin internal primer and gene specific primers listed in Table 2. For complementation, the respective genes along with 600-bp upstream sequence were cloned between *Nco*I and *Xba*I site of plasmid pCH112 and transformed into the corresponding mutant strain [39]. Primers used for complementation have been listed in Table 2.

**Table 2:** Primers used in this study

Primers	Sequence (5′–3′)
FwKoSEnWbaV	TAA GCATTTTTTA TACTATAAACTCGATTTGTATTGATGT TGATAT GTGTAGGCTG GAGCTGCTTC
RwKoSEnWbaV	GATTGCCAGTGTATTATATGTATAAGAGTAAAAGGCTAGC
	ATAATATATGAATATCCTCCTTAGTT
ConfKoSEnRfV	CAACCCCGTCCATACATATTATAGATACAT
FwKoSEnPrt	CC ACATCCACCG GTAATTAAAA GCT TCATTTCCCT TCCT C TTCAAT GTGTAGGCTG GAGCTGCTTC
RwKoSEnPrt	TAAATTTCTAATTTTTGAGGGGGGGGGATTCCCCTCTAT
	GATTCATATGAATATCCTCCTTAGTT
ConfKoSEnPrt	GCGAATATCACCATGTACAAACTC
FwKoSEnTyv	TTCTT TAATTATGCC CGCTTTCGCG GGCAGAAACA TCATA TAGAA T GTGTAGGCTGGAGCTGCTTC
RwKoSEnTyv	TTAACTGAAATAATTGAAGAGGAAGGGAAAATGAAGCTTT
	TAATTATATGAATATCCTCCTTAGTT
ConfKoSEnTyv	GGAATTCTAACCAACCTCAGTTTCCTC
ConfKt	CGGTCCGCCACACCCAGCC
FwCompwbaV	GTCGTTAGCCATGGGCCCATTATATATATATCGTTTC
RwCompwbaV	GGCGGGCGGTCTAGA CTATGAAAATATTTTTTTTATTACC
FwCompprt	GCGCGTTAA CCATGG ATTGTTATTGTGCGCCAGG
RwCompprt	GGCGGGCGGTCTAGA TCATTTCCCTTCCTCTTC
FwComptyv	GCGCGTTAA CCATGG AATAGAAAGCAATATTCTTATGC
RwComptyv	GGCGGGCGGTCTAGA TCATATAGAACTAGTCCAATC

### LPS analysis

LPS was prepared as described previously [40]. Briefly, bacterial cultures collected from LB agar plate were suspended in phosphate-buffered saline (PBS) with the help of cotton swab and optical density at 600 nm was adjusted to 2.0. From this 1.5 ml suspension was centrifuged and the pellet was resuspended in lysis buffer and boiled at 100 °C for 10 min followed by treatment with proteinase K enzyme and extraction in hot phenol. Final extraction of the aqueous phase was carried out with Tris-saturated ether. LPS preparations were resolved on 14 % acrylamide gels using Tricine-SDS buffer system and visualized by silver staining method as described earlier [40].

### Motility assay

Motility of wild-type *S.* Enteritidis and its isogenic mutants was analyzed by soft agar assay. Briefly, 1 µl of overnight grown culture of *S.* Enteritidis wild-type and its isogenic mutants was placed at the centre of the soft agar (0.3 % agar) plates and incubated at 37 °C. The diameter of the halo zone was measured after 4 h and 8 h. Plates were photographed with Nikon camera COOLPIXL810. Experiments were performed in triplicates and repeated thrice.

### Analysis of resistance to antimicrobial peptides

Overnight grown culture of wild-type and mutant strains was subcultured for 4 h. Cells were washed with PBS and diluted to approximately 10^8^ CFU/ml. A 50 µl aliquot of bacterial suspension was incubated at 37 °C for 1 h with or without AMPs, polymyxin B; 1 µg/ml (Sigma), protamine; 5 µg/ml (Himedia, India), LL37; 10 µg/ml (Sigma), cecropin; 2 µg/ml (Sigma) in PBS. Subsequently, samples were serially diluted and plated on LB agar plates to obtain the number of surviving bacteria. The survival efficiency was calculated as percent ratio of the CFU obtained from AMP treated culture versus untreated culture. Experiment was repeated thrice in triplicates.

### Analysis of serum resistance

Human serum samples, devoid of antibody against *Salmonella,* were obtained from healthy volunteers and pooled, divided into small aliquots and stored at −80 °C. Bacterial cultures grown overnight in LB medium were subcultured for 4 h. Cells were washed with PBS and diluted to approximately 10^8^ CFU/ml. 50 µl of diluted cell suspension was incubated with 50 µl of serum at 37 °C for 1 h. To determine the survival percentage, equal bacterial densities were incubated without serum under similar conditions. Number of surviving bacteria was evaluated by plating serial dilutions on LB agar plate. Survival percentage was calculated by dividing the number of bacteria survived with serum by the number of bacteria obtained from untreated culture. Experiment was performed thrice in triplicates.

### Attachment and invasion assay

For attachment and invasion assay, human colonic epithelial cell line (HCT-116) was cultured in Roswell Park Memorial Institute Medium (RPMI 1640) (HiMedia, India) supplemented with 10 % fetal bovine serum (FBS) (HiMedia, India). Cells were seeded on 24 well plates at the density of 2 × 10^5^ cells 16 h prior to infection. For infection, bacterial cultures were grown overnight under SPI1 inducing environment at 120 rpm. Overnight grown culture was diluted to 1:20 in fresh LB and subcultured without antibiotics for 4 h in SPI1 inducing condition. Bacterial cells were washed with PBS and inoculum was prepared in RPMI without antibiotics such that each well received 1 × 10^6^ bacterial cells to achieve a multiplication of infection (MOI) equal to 10. For the attachment assay, both inoculum and cells were prechilled on ice for 15 min to prevent the invasion. Infection was carried out on ice and further incubated for 30 min at 4 °C. Invasion assay was performed as described earlier [41]. Briefly, after 50 min of infection at room temperature, media was replaced with fresh infection media containing 100 µg/ml gentamicin to kill extracellular bacteria and incubated at 37 °C for 2 h in CO_2_ incubator (5 %). For both the assays, cells were lysed with 0.1 % sodium deoxycholate and appropriate dilutions were plated on LB agar plates supplemented with appropriate antibiotic/s to obtain the adherent and invasive bacteria. The percent of adherent/invasive bacteria was calculated by dividing the number of adherent/invasive bacteria by the number of infecting bacteria and multiplying by 100. Both, adhesion and invasion assays were performed thrice in triplicates.

### Mice infection experiments

All animals (C57BL/6 mice) used for the experiments were specific pathogen free (SPF). This animal model is used because of its susceptibility to many broad and narrow host range *Salmonella* species [42]. Mice were purchased and maintained at the animal house facility of KIIT School of Biotechnology, Odisha, India. 6–8 weeks old specific pathogen free mice were used. All experiments were performed in according to the guidelines of the Institutional Animal Ethics Committee (IAEC), KIIT University, under approval number: KSBT/IAEC/2013/MEET1/A6. During the experiments, all mice were maintained in individually ventilated cages. All the animal experiments were performed in strict accordance with guidelines laid by the Institutional Animal Ethics Committee (IAEC). The infection experiments were carried out in streptomycin pre-treated mice models as established previously [43]. Briefly, a group of five mice were infected (by gavage) with approximately 10^7^ CFU of wild-type, Δ*wbaV* and Δ*prt* mutants of *S.* Enteritidis. The bacterial loads in the cecum content, mesenteric lymph node (mLN), spleen and liver were determined at 3 days p. i. by plating suitable dilutions of tissue homogenates on MacConkey agar supplemented with the appropriate antibiotics. For statistical analysis, samples without bacterial counts were adjusted to the minimal detection level (20 CFU/organ in the spleen and 60 CFU/organ in liver). Cecal inflammation of the infected mice was scored to analyze the degree of inflammation.

### Histopathological evaluation

Segments of the cecum, colon and ileum were embedded in O.C.T. (Sakura Finetek Inc., USA), snap-frozen in liquid nitrogen, and stored at −80 °C. The 5 μm thick tissue sections were obtained on glass slides and stained with hematoxylin and eosin (H&E) after drying for at least 2 h at room temperature. The stained cryosections were evaluated on the basis of a previously described scoring system for the quantitative analysis of cecal inflammation [43, 44]. The stained sections were scored on the basis of the pathological changes that include sub-mucosal edema, polymorphonuclear leukocyte (PMN) infiltration, loss of goblet cells and epithelial ulceration. The collective pathological score ranges from 0 to 13 with arbitrary units covering the inflammation levels that included intact intestine without any sign of inflammation (pathoscore 0); least sign of inflammation (pathoscore 1–2), slight inflammation as a minimal sign of tissue pathology (pathoscore 3–4); moderate inflammation (pathoscore 5–8); and significant inflammation (pathoscore 9–13).

### Statistical analysis

All the in vitro data represent mean ± standard deviation of three independent experiments. Two-way analysis of variance (ANOVA) was employed to determine significant differences in case of sensitivity to different antimicrobial peptides whereas in remaining experiments *t* test was used. All the statistical calculations were performed with the help of GraphPad Prism software version 5.

## Abbreviations

*S*. Enteritidis: *Salmonella enterica* serovar Enteritidis
PBS: phosphate buffered saline
LPS: lipopolysaccharide
OAg: O-antigen
mLN: mesenteric lymph node
AMP: antimicrobial peptide
